# Association between Osteocalcin, Metabolic Syndrome, and Cardiovascular Risk Factors: Role of Total and Undercarboxylated Osteocalcin in Patients with Type 2 Diabetes

**DOI:** 10.1155/2013/197519

**Published:** 2013-04-09

**Authors:** Assim A. Alfadda, Afshan Masood, Shaffi Ahamed Shaik, Hafedh Dekhil, Michael Goran

**Affiliations:** ^1^Obesity Research Center, College of Medicine, King Saud University, P.O. Box 2925 (98), Riyadh 11461, Saudi Arabia; ^2^Department of Medicine, College of Medicine, King Saud University, P.O. Box 2925 (38), Riyadh 11461, Saudi Arabia; ^3^Department of Family and Community Medicine, College of Medicine, King Saud University, P.O. Box 2925, Riyadh 11461, Saudi Arabia; ^4^Department of Preventive Medicine, University of Southern California, Los Angeles, CA 90089, USA

## Abstract

Studies have demonstrated that total osteocalcin (TOC) is associated with metabolic syndrome (MetS) and therefore might influence the risk of cardiovascular disease in humans. Undercarboxylated osteocalcin (uOC) regulates insulin secretion and sensitivity in mice, but its relation to MetS in humans is unclear. We aimed to determine whether uOC is related to MetS and/or its individual components and other cardiovascular risk factors in patients with type 2 diabetes mellitus (T2DM), and whether TOC and uOC have utility in predicting the cardiovascular risk. We studied 203 T2DM patients with and without MetS. MetS was defined based on the NCEP-ATP III criteria. A correlation analysis was performed between the three outcome variables: (i) TOC, (ii) uOC, and (iii) carboxylated osteocalcin (cOC) and MetS components and other cardiovascular risk factors. Both TOC and uOC were significantly lower in patients with MetS compared to those without MetS, independent of body mass index. In patients with MetS, uOC was significantly and positively correlated with HDL cholesterol, while TOC was significantly and negatively correlated with serum triglycerides. We report for the first time that uOC is related to lipid indices in patients with T2DM. Further studies are necessary to determine whether uOC can be utilized for cardiovascular risk assessments in these patients.

## 1. Introduction

Metabolic syndrome (MetS) is a conglomeration of abnormalities relating metabolism to cardiovascular risk. Factors involved with this syndrome include atherogenic dyslipidemias, central obesity, increased blood pressure, and impaired fasting glucose levels. Individuals diagnosed with MetS have a 5-fold risk for type 2 diabetes mellitus (T2DM) and a 2-fold risk for cardiovascular disease (CVD) [1]. Risk factors for MetS are concordant with each other and have been defined by many groups. The National Cholesterol Education Program-Adult Treatment Panel III (NCEP-ATP III) guidelines have identified MetS as a multiplex risk factor for CVD that deserves more clinical attention.

The skeleton, which is typically considered a static structure that is important for providing the necessary framework of the body, has recently been found to play an important role in the maintenance of energy homeostasis. The bones, adipose tissue, and brain interact with one another to control body weight and to regulate energy expenditure and glucose metabolism through the actions of a hormone that is secreted specifically by the osteoblasts, namely, osteocalcin [2, 3]. Osteocalcin (OC), or bone Gla protein, is the major noncollagenous protein that acts locally in the bone mineralization. OC is synthesized by the osteoblasts as the pre-pro-form and is cleaved to the mature protein with subsequent enzymatic posttranslational carboxylation [4]. This molecule is present in the circulation in the carboxylated (cOC) form and, to a lesser degree, in the undercarboxylated (uOC) form. Total serum OC (TOC), which comprises both the carboxylated and the undercarboxylated forms, has been utilized traditionally as a marker of bone formation or bone turnover.

Studies using animal models have demonstrated that OC is involved in the regulation of body energy by modulating fat and glucose metabolism [5]. The uOC acts directly on the *β*-cells to increase their mass and proliferation and therefore increases insulin secretion. Moreover, it influences white adipocytes to induce the expression of genes involved in energy expenditure and to enhance the secretion of adiponectin, thus increasing insulin sensitivity [6]. Apart from its secretion from the osteoblasts, OC is also secreted locally by adipocytes and megakaryocytes [7, 8]. Combining these actions, OC is capable of enhancing the secretion of insulin and increasing insulin sensitivity in both fat and muscle.

Consistent with the animal studies, OC levels have been reported to be decreased in patients with T2DM and to be inversely related to the levels of insulin resistance and adiposity in humans [9, 10]. Cross-sectional analyses have extended these observations to apply the MetS criteria in examining the association between OC level and cardiovascular risk; the results have revealed that OC is inversely related to the number of MetS components [1, 2]. Many of these studies have used TOC to study this relationship. Shea et al. reported that the carboxylated fraction is inversely related to impaired glucose metabolism indices [11]. By contrast, Kanazawa et al. demonstrated that serum uOC and TOC levels are inversely related to impaired glucose levels [12]. The same group also found that the uOC/TOC ratio is not related to the fasting plasma glucose or the diabetic status. In their FORMEN study, Iki et al. reported that the total, carboxylated and undercarboxylated forms are all proportional to one another [13]. Considering these studies in combination, it appears that the role of the various OC fractions in humans is still unclear.

To date, one of the main limitations of the studies on this subject is that they have measured one or two fractions of OC, and no study has measured all three fractions together. Because prior studies have demonstrated that the undercarboxylated fraction is implicated in the pathophysiology of insulin sensitivity, further investigations in this area should include a direct measurement of this fraction. In an attempt to fill this gap, we measured the carboxylated, undercarboxylated, and total OC in a population of patients with T2DM who were either positive or negative for MetS. Our objective was to determine whether uOC is related to MetS and/or its individual components and other cardiovascular risk factors in patients with T2DM, and whether TOC and uOC have utility in predicting cardiovascular risk.

## 2. Materials and Methods

In this cross-sectional study, 203 consecutive patients with T2DM followed regularly at a diabetes outpatient clinic were studied. All the patients were ≥40 years of age, were not taking any lipid-lowering drugs, and had serum triglyceride levels of <3.38 mmol/L. The exclusion criteria included the presence of any significant liver disease, renal failure requiring renal-replacement therapy, and the usage of oral contraceptives, hormone replacement therapy, or other drugs that could influence bone metabolism. This study was performed at the Obesity Research Center, College of Medicine, King Saud University, Riyadh, Saudi Arabia. The Institutional Review Board approved this study, and all the participants gave their informed consent.

Anthropometric measurements (including height and weight) were taken, and the body mass index (BMI) was calculated. The waist circumference was determined as the minimum value between the iliac crest and the lateral costal margin.

Serum TOC was measured using an N-MID Osteocalcin ELISA kit (Elecsys, Roche diagnostic Ltd., Switzerland). The serum uOC and serum cOC were measured using EIA kits (Takara Bio Inc., Japan). The undercarboxylated EIA kit uses a set of monoclonal antibodies that are reactive to uOC and less reactive to cOC at amino acid positions 17, 21, and 24. The carboxylated EIA kit uses a set of monoclonal antibodies that are reactive to cOC at amino acid position 17. The intraassay and inter-assay variabilities were, respectively, 5.7% and 6.2% for cOC, 10.2% and 9.8% for uOC, and 3.4% and 3.6% for TOC. The total cholesterol and triglycerides were determined by enzymatic techniques, whereas apolipoprotein A-1 (Apo A-1) and apolipoprotein B (Apo B) were determined by immune-turbidimetric assays (Kone Instruments, Espoo, Finland). HDL cholesterol was measured after precipitation of VLDL and LDL with phosphotungstic acid and magnesium chloride. LDL cholesterol was calculated using Friedewald's equation [14].

We used the definition of MetS according to the NCEP-ATP III. Within this definition, three or more of the following criteria must be fulfilled: fasting blood glucose level ≥5.6 mmol/L, blood pressure ≥130/85 mmHg, triglycerides ≥1.7 mmol/L, HDL cholesterol <1.03 mmol/L for men and <1.29 mmol/L for women, and waist circumference >102 cm for men and >88 cm for women [15].

## 3. Statistical Analysis

The statistical analysis was performed using SPSS version 17 (Chicago, Illinois, USA). All the data are presented either as the mean ± SD (for symmetric variables) or median with inter quartile range (for skewed variables). Independent Student's *t*-test and Mann-Whitney *U* test were used to compare the means and mean ranks between the groups. Kruskal-Wallis test was used to compare the mean ranks of TOC, uOC, and cOC across the three groups of MetS components. Logarithmic transformations for skewed outcome variables (TOC, uOC, and cOC) were done so as to carry out the regression analysis. Karl Pearson's correlation analysis was performed to determine the relationships between two continuous variables of interest. A multiple linear regression analysis was performed to test the independent linear relationship between the logarithmic transformed serum OC level and the markers of lipid metabolism by adjusting the effect of confounding variables, including age, BMI, waist circumference, and HbA1c. The results were considered statistically significant when *P* < 0.05.

## 4. Results

### 4.1. Baseline Characteristics

The characteristics of the study population are listed in Table 1. All the studied patients were affected by T2DM and either overweight or obese. The levels of the three different forms of OC (Table 1) were lower than what has been reported in normal individuals [1]. The three different forms of OC were measured independently and were significantly correlated with each other (*P* = 0.00, data not shown).

The measured TOC was inversely related to the markers of adiposity, as assessed via BMI (*r* = −0.16, *P* = 0.02) and glycemia (indicated by the HbA1c: *r* = −0.2, *P* = 0.01), as shown in Table 2. The serum uOC was significantly and positively correlated with HDL-cholesterol (*r* = 0.15, *P* = 0.03) and negatively correlated with Apo B/Apo A-1 ratio (*r* = −0.16, *P* = 0.02).

**Figure 1:**
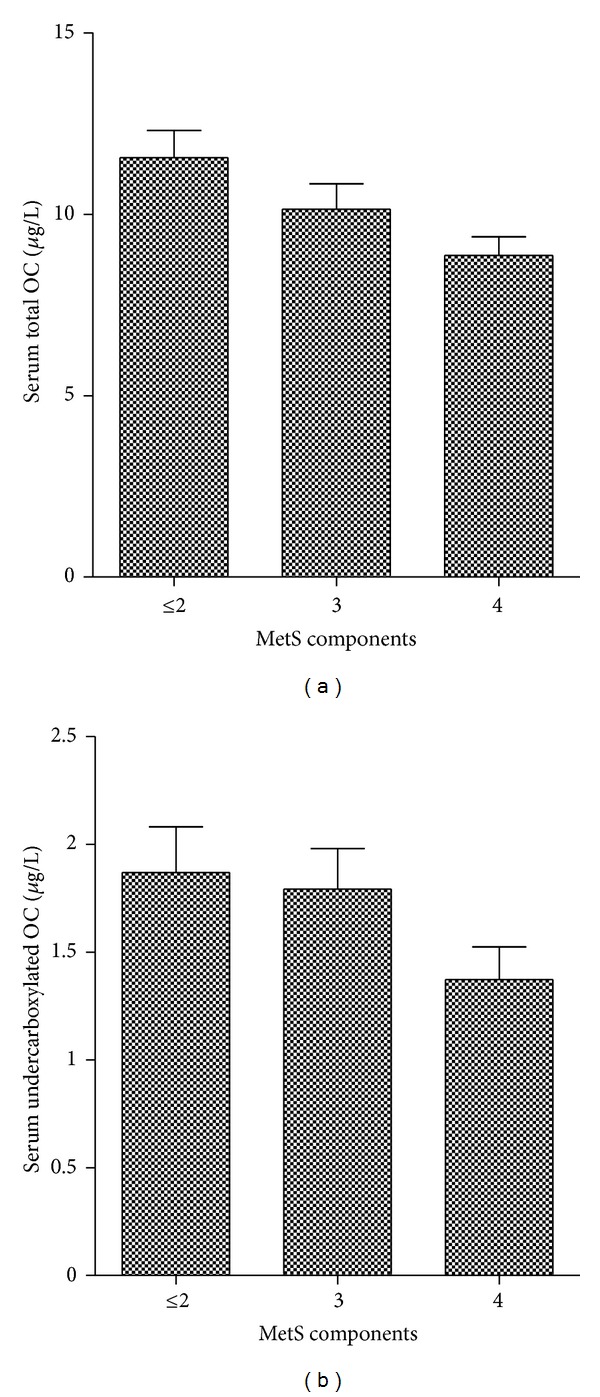
Serum total and undercarboxylated osteocalcin levels in relation to the number of metabolic syndrome components. Data are expressed as mean ± SEM. A significant decrease in total osteocalcin levels was observed with a higher number of metabolic syndrome components (*P* = 0.02). A Similar trend was observed with the undercarboxylated osteocalcin but this did not reach statistical significance (*P* = 0.11).

Subsequently, the study population was divided based on the presence or absence of MetS, according to the NCEP-ATP III criteria. MetS was diagnosed in 66% of the study population. Individuals with MetS, when compared to those subjects without MetS, exhibited significantly higher BMIs, waist circumferences, triglycerides levels, Apo B levels, and Apo B/Apo A-1 ratios (*P* = 0.00), as presented in Table 1. Individuals without MetS had higher levels of HDL-cholesterol (*P* = 0.00). The group with MetS had lower levels of TOC and uOC, compared with those subjects without MetS (*P* = 0.01 and *P* = 0.03, resp.). A significant decrease in TOC levels was observed with a higher number of MetS components (*P* = 0.02). A similar trend was observed with the uOCs but this did not reach statistical significance (Figure 1).

**Figure 2:**
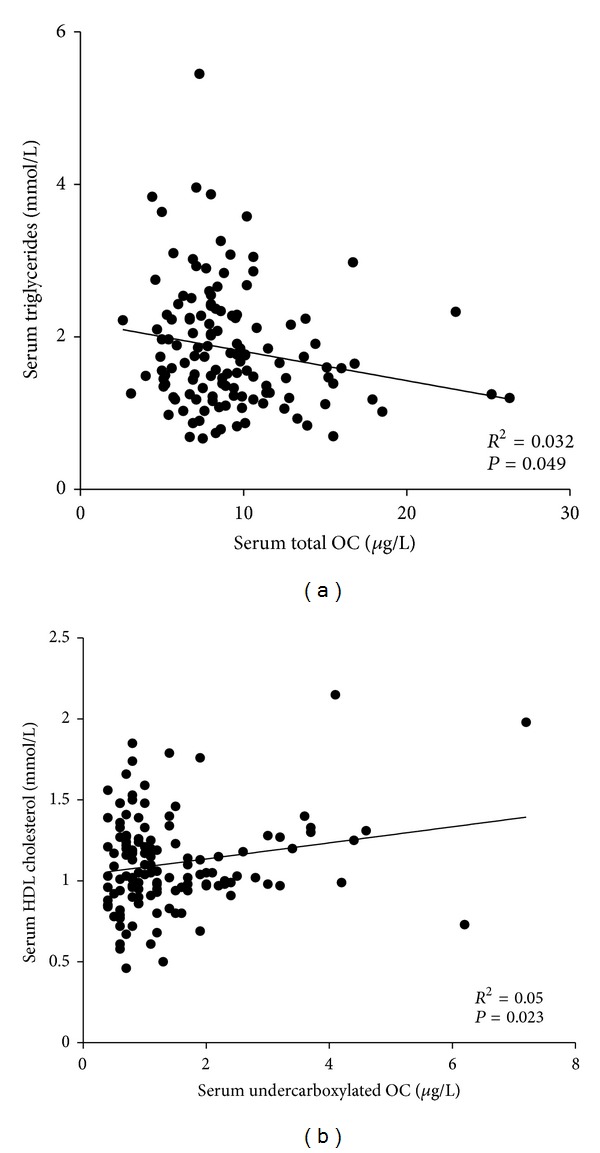
Scatter plot and regression analysis of serum TOC and uOC levels with serum triglycerides and serum HDL-cholesterol levels, respectively, within the group of individuals with the metabolic syndrome. The serum TOC was significantly and negatively correlated with serum triglycerides (*R* ^2^ = 0.032, *P* = 0.049), whereas uOC significantly correlated with serum HDL-cholesterol levels (*R* ^2^ = 0.05, *P* = 0.023).

To further investigate whether the serums TOC and uOC levels in patients with MetS were related to markers of dyslipidemia independently of other risk factors, multiple regression analyses adjusted for age, BMI, waist circumference, and HbA1c were performed between TOC and uOC versus the lipid parameters. The serum TOC was significantly and negatively correlated with serum triglycerides (*R* ^2^ = 0.032, *P* = 0.049), whereas uOC significantly correlated with serum HDL-cholesterol levels (*R* ^2^ = 0.05, *P* = 0.023) as shown in Figure 2. 

## 5. Discussion

To our knowledge, the present study is the first to measure all the different forms of OC. For the first time, we established an association between the uOC fraction and the lipidemic status in individuals with MetS. In agreement with other studies, we found that TOC is related to the glycemic control and markers of adiposity in patients with T2DM.

Previous studies have demonstrated that TOC levels are significantly and negatively correlated with the fasting blood glucose and HbA1c levels [10, 16, 17]. An inverse association between the TOC level and HbA1c was also observed in the present study. This finding indicates that among our patients with T2DM those individuals with better glycemic control are more likely to exhibit higher TOC levels. This result would also indicate that prolonged hyperglycemia and uncontrolled diabetes negatively affect the TOC level. By contrast, we did not determine an association between uOC and the measures of glycemia. It is possible that the antidiabetic medications received by our patients could affect the levels of uOC; thus, further studies are necessary to clarify this point.

In our study, TOC was significantly and negatively correlated with BMI. This finding confirms other investigations demonstrating that TOC is negatively associated with BMI and fat mass [16]. There was no correlation of uOC with BMI in our cohort.

The uOC was significantly and positively correlated with HDL-cholesterol but was negatively correlated with the Apo B/Apo A-1 ratio. This finding confirms a prior report of Lee et al., who demonstrated this relationship in animals [5]. To our knowledge, the present study is the first to establish a relationship between uOC and the Apo B/Apo A-1 ratio in humans, suggesting that a low uOC is closely related to an atherogenic, dyslipidemic profile that would otherwise increase the cardiovascular risk. Individuals with T2DM are known to exhibit a classic lipid triad that includes high triglyceride levels, low HDL-cholesterol levels, and normal LDL-cholesterol levels. However, this triad is not sufficient to predict an untoward cardiovascular event. The Apo B/Apo A-1 ratio is superior to any other cholesterol measurement for predicting cardiovascular risk [18–20]. As determined in a large prospective AMORIS study, higher Apo B levels, an increased Apo B/Apo A-1 ratio, and low levels of Apo A-1 are highly predictive of risk of fatal myocardial infarction [19, 21]. The inverse association of uOC with the ApoB/Apo A-1 ratio that was reported in our study suggests the possible role of uOC in cardiovascular risk assessment in patients with T2DM. Although it would be premature to conclude a causal effect of uOC on these parameters, it would be of interest to explore whether interventions that specifically raise uOC levels would decrease cardiovascular risk.

MetS is a collection of abnormalities that increases the likelihood of CVD. The goal of identifying MetS is to prevent the occurrence of these diseases. To investigate whether the presence of MetS increases the relationship between uOC and the prediction of CVD risk, we divided our population (based on the NCEP-ATP III guidelines) into those subjects with and those without MetS. As expected, there were significant differences between the two groups. Patients with MetS had a higher BMI, waist circumference, triglyceride level, Apo B level and Apo B/Apo A-1 ratio, and lower HDL-cholesterol level. We also found that the TOC and the uOC, but not the cOC, were significantly lower in patients with MetS in comparison to those without MetS. These differences point toward an association between these forms of OC and the dyslipidemia that is found in patients with MetS. Previous studies mainly focused on determining the role of circulating TOC, but they did not differentiate between the different forms of OC. To resolve this issue, we measured all the OC fractions independently, thus overcoming the problem associated with either calculating the cOC levels by subtracting the uOC from the TOC or adding the cOC and uOC fractions to obtain the total OC, as has been done by Polgreen et al. [22]. In our study, we found that the measured TOC value was higher than the value obtained by summing up the two individual fractions (data not shown). To study how the uOC and TOC are associated with CVD risk, we compared the relationship between the MetS components and other cardiovascular risk factors and the different forms of OC. The cOC fraction did not exhibit any relationship with the MetS components. There was an inverse association between the TOC and the waist circumference values and serum triglycerides levels, although the former did not reach statistical significance. Our findings confirm recent studies demonstrating that serum TOC levels are associated with MetS. Saleem et al. determined that serum TOC is negatively associated with MetS in both blacks and non-Hispanic whites [2]. Moreover, Yeap et al. reported that men with lower serum TOC concentrations have a higher risk of MetS [23]. Oosterwerff et al. found that plasma OC was inversely associated with MetS in a community-dwelling cohort of older persons in the Netherlands; the authors reported that the subjects with the lowest quartile of OC concentrations had an approximately 3.7-fold higher risk of MetS than did the subjects with the highest quartile [24].

Our study had several limitations. First, the study population is comprised of subjects who were followed for T2DM at a tertiary care hospital and thus might not have been representative of the general population. Second, this cross-sectional study could not determine a causal relationship between the fractions of OC and MetS. Third, we did not measure sex steroids and vitamin K intake which could influence OC and bone metabolism.

## 6. Conclusions

The present study measured the three independent forms of OC in patients with T2DM. We found that the TOC level was associated with the degree of glycemic control, independently of the carboxylated or undercarboxylated forms. We have established a new relationship between the uOC and lipid indices (particularly the HDL-cholesterol and Apo B/ApoA-1 ratio), and we therefore suggest that uOC could play a role in the evaluation of the cardiovascular risk in patients with T2DM. If serum osteocalcin is employed to determine the cardiovascular risk in patients with T2DM, then the measured uOC would be a better predictor than the TOC. Nonetheless, prospective studies are necessary to investigate further the utility of uOC for cardiovascular risk prediction.

## Figures and Tables

**Table 1:** Clinical and biochemical characteristics of the study population in all groups and based on the presence or absence of metabolic syndrome.

Variable	All	MetS	Non-MetS	*P*
Number (*n*)	203	134	69	
Age (years)	52.5 ± 9.6	52.3 ± 9.7	52.7 ± 9.1	0.74
BMI (kg/m^2^)	30.8 ± 5.7	32.2 ± 5.5	28.05 ± 5.3	**0.00**
Waist circumference	100.7 ± 12.7	104.5 ± 10.8	92 ± 12.25	**0.00**
HbA1c	8.8 ± 1.9	8.6 ± 1.8	8.38 ± 2.13	0.38
Total cholesterol (mmol/L)	5.0 ± 0.9	5.1 ± 0.94	4.9 ± 0.92	0.1
LDL-cholesterol (mmol/L)	3.1 ± 0.8	3.1 ± 0.88	3.0 ± 0.75	0.29
HDL-cholesterol (mmol/L)	1.1 ± 0.3	1.09 ± 0.31	1.29 ± 0.38	**0.00**
Triglycerides (mmol/L)	1.6 ± 0.7	1.83 ± 0.78	1.26 ± 0.52	**0.00**
Apo B (g/L)	1.0 ± 0.2	1.14 ± 0.26	0.99 ± 0.21	**0.00**
Apo A-1 (g/L)	1.3 ± 0.2	1.35 ± 0.24	1.41 ± 0.31	0.17
Apo B/Apo A-1	0.8 ± 0.2	0.85 ± 0.22	0.74 ± 0.28	**0.00**

	Median (IQR)	MetS	Non-MetS	P*

Total OC (*μ*g/L)	8.8 (4.5)	8.4 (3.7)	9.8 (5.8)	**0.01**
Carboxylated OC (*μ*g/L)	0.53 (0.5)	0.54 (0.6)	0.53 (0.4)	0.66
Undercarboxylated OC (*μ*g/L)	1.14 (1.1)	1.04 (1.0)	1.4 (1.7)	**0.03**

Data are presented as the mean ± standard deviation unless otherwise stated. MetS: metabolic syndrome, BMI: body mass index, LDL: low-density lipoprotein, HDL: high-density lipoprotein, Apo A-1: apolipoprotein A-1, Apo B: apolipoprotein B, OC: osteocalcin. The *P* values were yielded from the MetS versus Non-MetS comparison of the values for each of the measured parameters. *P* values < 0.05 (bold) were considered statistically significant. IQR: interquartile range. *Mann-Whitney *U* test.

**Table 2:** Analysis of the overall correlation between the biochemical variables and the total and undercarboxylated osteocalcin.

Variable	Total osteocalcin		Undercarboxylated osteocalcin		Carboxylated osteocalcin	
	*r*	*P*	*r*	*P*	*r*	*P*
Age	0.09	0.22	0.06	0.4	0.33	**0.00**
BMI	−0.16	**0.02**	−0.02	0.74	0.005	0.95
HbA1c	−0.20	**0.01**	−0.09	0.25	−0.12	0.12
HDL-cholesterol	0.03	0.71	0.15	**0.03**	−0.06	0.41
Apo A-1	−0.01	0.85	0.12	0.09	−0.06	0.38
Apo B/Apo A-1	−0.12	0.09	−0.16	**0.02**	0.12	0.09

HDL: high-density lipoprotein, Apo A-1: apolipoprotein A-1, Apo B: apolipoprotein B. *P* values < 0.05 (bold) were considered statistically significant.

## References

[1] Tan A, Gao Y, Yang X (2011). Low serum osteocalcin level is a potential marker for metabolic syndrome: results from a Chinese male population survey. *Metabolism*.

[2] Saleem U, Mosley TH, Kullo IJ (2010). Serum osteocalcin is associated with measures of insulin resistance, adipokine levels, and the presence of metabolic syndrome. *Arteriosclerosis, Thrombosis, and Vascular Biology*.

[3] Gomez-Ambrosi J, Rodriguez A, Catalan V (2008). The bone-adipose axis in obesity and weight loss. *Obesity Surgery*.

[4] Lee AJ, Hodges S, Eastell R (2000). Measurement of osteocalcin. *Annals of Clinical Biochemistry*.

[5] Lee NK, Sowa H, Hinoi E (2007). Endocrine regulation of energy metabolism by the skeleton. *Cell*.

[6] Ferron M, Hinoi E, Karsenty G, Ducy P (2008). Osteocalcin differentially regulates *β* cell and adipocyte gene expression and affects the development of metabolic diseases in wild-type mice. *Proceedings of the National Academy of Sciences of the United States of America*.

[7] Benayahu D, Shamay A, Wientroub S (1997). Osteocalcin (BGP), gene expression, and protein production by marrow stromal adipocytes. *Biochemical and Biophysical Research Communications*.

[8] Thiede MA, Smock SL, Petersen DN, Grasser WA, Thompson DD, Nishimoto SK (1994). Presence of messenger ribonucleic acid encoding osteocalcin, a marker of bone turnover, in bone marrow megakaryocytes and peripheral blood platelets. *Endocrinology*.

[9] Kim SH, Lee JW, Im JA, Hwang HJ (2010). Serum osteocalcin is related to abdominal obesity in Korean obese and overweight men. *Clinica Chimica Acta*.

[10] Kindblom JM, Ohlsson C, Ljunggren O (2009). Plasma osteocalcin is inversely related to fat mass and plasma glucose in elderly Swedish men. *Journal of Bone and Mineral Research*.

[11] Shea MK, Gundberg CM, Meigs JB (2009). *γ*-carboxylation of osteocalcin and insulin resistance in older men and women. *American Journal of Clinical Nutrition*.

[12] Kanazawa I, Yamaguchi T, Yamauchi M (2011). Serum undercarboxylated osteocalcin was inversely associated with plasma glucose level and fat mass in type 2 diabetes mellitus. *Osteoporosis International*.

[13] Iki M, Tamaki J, Fujita Y (2012). Serum undercarboxylated osteocalcin levels are inversely associated with glycemic status and insulin resistance in an elderly Japanese male population: fujiwara-kyo Osteoporosis Risk in Men (FORMEN) Study. *Osteoporosis International*.

[14] Friedewald WT, Levy RI, Fredrickson DS (1972). Estimation of the concentration of low-density lipoprotein cholesterol in plasma, without use of the preparative ultracentrifuge. *Clinical Chemistry*.

[15] Grundy SM (2001). United States cholesterol guidelines 2001: expanded scope of intensive low-density lipoprotein-lowering therapy. *American Journal of Cardiology*.

[16] Kanazawa I, Yamaguchi T, Tada Y, Yamauchi M, Yano S, Sugimoto T (2011). Serum osteocalcin level is positively associated with insulin sensitivity and secretion in patients with type 2 diabetes. *Bone*.

[17] Pittas AG, Harris SS, Eliades M (2009). Association between serum osteocalcin and markers of metabolic phenotype. *Journal of Clinical Endocrinology & Metabolism*.

[18] Sniderman AD (2003). Non-HDL cholesterol versus apolipoprotein B in diabetic dyslipoproteinemia: alternatives and surrogates versus the real thing. *Diabetes Care*.

[19] Walldius G, Jungner I (2004). Apolipoprotein B and apolipoprotein A-I: risk indicators of coronary heart disease and targets for lipid-modifying therapy. *Journal of Internal Medicine*.

[20] Sierra-Johnson J, Somers VK, Kuniyoshi FHS (2006). Comparison of apolipoprotein-B/apolipoprotein-AI in subjects with versus without the metabolic syndrome. *American Journal of Cardiology*.

[21] Walldius G, Jungner I, Holme I, Aastveit AH, Kolar W, Steiner E (2001). High apolipoprotein B, low apolipoprotein A-I, and improvement in the prediction of fatal myocardial infarction (AMORIS study): a prospective study. *The Lancet*.

[22] Polgreen LE, Jacobs DR, Nathan BM (2012). Association of osteocalcin with obesity, insulin resistance, and cardiovascular risk factors in young adults. *Obesity*.

[23] Yeap BB, Chubb SA, Flicker L (2012). Associations of total osteocalcin with all-cause and cardiovascular mortality in older men. The Health In Men Study. *Osteoporosis International*.

[24] Oosterwerff MM, van Schoor NM, Lips P (2013). Osteocalcin as a predictor of the metabolic syndrome in older persons: a population-based study. *Clinical Endocrinology*.

